# Transcriptome Dynamics during Black and White Sesame (*Sesamum indicum* L.) Seed Development and Identification of Candidate Genes Associated with Black Pigmentation

**DOI:** 10.3390/genes11121399

**Published:** 2020-11-25

**Authors:** Linhai Wang, Senouwa Segla Koffi Dossou, Xin Wei, Yanxin Zhang, Donghua Li, Jingyin Yu, Xiurong Zhang

**Affiliations:** 1 Key Laboratory of Biology and Genetic Improvement of Oil Crops of the Ministry of Agriculture, Oil Crops Research Institute of the Chinese Academy of Agricultural Sciences, Wuhan 430062, China; linhai827@163.com (L.W.); dossouf@yahoo.fr (S.S.K.D.); 13277065857@163.com (Y.Z.); ldh360681@163.com (D.L.); yujingyin@caas.cn (J.Y.); 2 Shanghai Key Laboratory of Plant Molecular Sciences, College of Life Sciences, Shanghai Normal University, Shanghai 200234, China; xwei@shnu.edu.cn

**Keywords:** *Sesamum indicum*, seed coat color, transcriptome, candidate genes, black pigment

## Abstract

Seed coat color is a crucial agronomic trait in sesame (*Sesamum indicum L.*) since it is strongly linked to seed oil, proteins, and lignans contents, and also influences consumer preferences. In East Asia, black sesame seed is used in the treatment and the prevention of various diseases. However, in sesame, little is known about the establishment of the seed coat color, and only one gene has been reported to control black pigmentation. This study provides an overview of developing seeds transcriptome of two varieties of sesame “Zhongfengzhi No.1” (white seed) and “Zhongzhi No.33” (black seed) and shed light on genes involving in black seed formation. Until eight days post-anthesis (DPA), both the seeds of the two varieties were white. The black sesame seed turned to yellow between 9 and 11 DPA and then black between 12 and 14 DPA. The black and white sesame showed similar trend-expressed genes with the numbers increased at the early stages of seed development. The differentially expressed genes (DEGs) number increased with seed development in the two sesame varieties. We examined the DEGs and uncovered that more were up-regulated at the early stages. The DEGs between the black and white sesame were mainly enriched in 37 metabolic pathways, among which the flavonoid biosynthesis and biosynthesis of secondary metabolites were dominants. Furthermore, we identified 20 candidate genes associated with pigment biosynthesis in black sesame seed, among which 10 were flavonoid biosynthesis and regulatory genes. These genes also include isochorismate and polyphenol oxidase genes. By comparing the phenotypes and genes expressions of the black and white sesame seed at different development stages, this work revealed the important role of 8–14 DPA in black pigment biosynthesis and accumulation. Moreover, it unfolded candidate genes associated with black pigmentation in sesame. These findings provide a vast transcriptome dataset and list of genes that will be targeted for functional studies related to the molecular mechanism involved in biosynthesis and regulation of seed coat color in sesame.

## 1. Introduction

Sesame (*Sesamum indicum L.*), owned to the Pedaliaceae family, is a vital oil crop widely grown in tropical, subtropical, and southern temperate regions due to its high-quality nutritional and therapeutic seeds. Sesame is an ideal plant model for studying many traits related to the oil content in crops because of its diploid nature and tiny genome size of 357Mb [1]. Moreover, it exhibits contrasting genetic dissimilarity in morpho-economic traits and quality attributes (seed shape, color, length) [2]. A high polymorphism was observed with the seed coat color [3]. Sesame seed color generally varies from black, brown, and yellow, to white. Previous studies revealed that white sesame seeds contained higher sesamin and sesamolin [4,5], oil, protein, and linoleic acid [6]. In contrast, black sesame seeds generally possess a high level of ash and carbohydrate [7], total phenolics, and total antioxidants [8]. Due to the various health-promoting abilities of natural antioxidants, medical and pharmaceutical industry researchers have been engaged to develop food products with a high amount of antioxidants principally for nutritional goals [9,10,11]. Black sesame products attract good acceptance in East Asia and are used in the treatment of diverse diseases due to their high antioxidative ability [12]. In contrast, white sesame seeds are primarily used to produce oil and as food.

**Figure 1:**
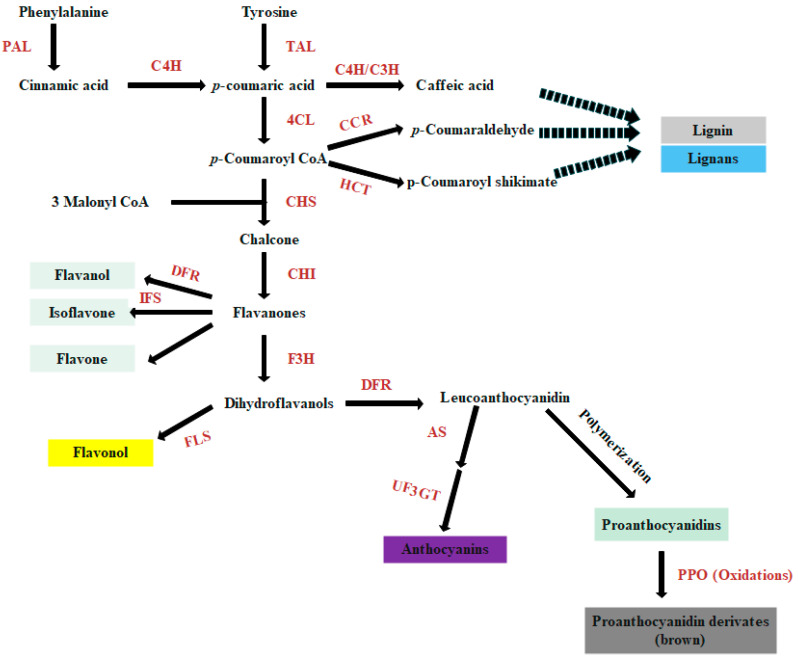
A diagram of the general biosynthetic pathway of phenylpropanoid leading to the main subgroups, and including lignin and flavonoids. Key enzymes catalyzing some reactions: PAL, phenylalanine amonialyase; C4H, cinnamate 4-hydroxylase; 4CL, 4 coumaroyl-coenzyme A ligase; CHS, chalcone synthase; CHI, chalcone isomerase; F3H, flavanone 3′-hydroxylase; DFR, dihydroflavonol 4-reductase; FLS, flavonol synthase; IFS, isoflavonoid synthase; AS, anthocianin synthase; UF3GT, UDP glucose: flavonoid 3-O-glucosyltransferase; TAL, Tyrosine ammonia-lyase; C3H, p-Coumarate 3-hydroxylase; CCR, Cinnamoyl-CoA reductase; HCT, Hydroxycinnamoyl-CoA shikimate/quinatehydroxycinnoyltransferase; and PPO, Polyphenol oxidase. Adapted from ref [16,18,19].

The seed coat not only defends the developing zygote against adverse external factors but also helps the seed to adjust its metabolism in response to environmental changes [13]. Seed coat color is a complex polygenetic trait. It has been reported to involve several pigments, including flavonoids, lignin, and melanin [14]. Shoeva and colleagues [15] reported that flavonoids are responsible for yellow, purple, and blue barley grain while black barley pigmentation is caused by melanins, oxidized and polymerized phenolic compounds. According to their chemical structure, flavonoids are divided into many subclasses, including flavones, flavonols, flavanones, anthocyanins, isoflavonoids, flavan-3-ols, and proanthocyanidins [16,17]. Flavonoids and lignin are biosynthesized from phenylalanine via the general phenylpropanoid pathway (Figure 1) [16,18,19]. The structural genes involved in flavonoids biosynthesis have been well characterized and described [18,20]. The main functional and regulatory genes include chalcone synthase (CHS), chalcone isomerase (CHI), flavonol 3-hydroxylase (F3H), flavonol 3’-hydroxylase (F3H), dihydroflavonol-4-reductase (DFR), anthocyanidin synthase (ANS), and anthocyanidin reductase (ANR). The regulatory proteins; MYBs, bHLH (basic helix-loop-helix), and WDR transcription factors of flavonoid biosynthesis were also identified [21]. The biosynthesis of lignin occurs in plants from oxidative polymerization of monolignols *p*-coumaryl, coniferyl, and sinapyl alcohols [19]. The melanogenesis in plants is associated with the browning reaction that is controlled genetically by many genes, among which the genes encoding polyphenol oxidases (PPO) might be principal [22].

Several years ago, sesame research entered the “Omics” era [23]. Since then, a list of QTL and candidate genes have been identified related to oil, sesamin and sesamolin content, fatty acid biosynthesis and yield, plant height and seed coat color [1,24,25,26]. Zhang et al. [27] have detected four QTL (QTL1-1, QTL11-1, QTL11-2, and QTL13-1) for seed coat color, whereas Wang et al. [25] have detected nine QTL (qSCa-8.2, qSCb-4.1, qSCb-8.1, qSCb-11.1, qSCl-4.1, qSCl-8.1, qSCl-11.1, qSCa-4.1, and qSCa-8.1) located on chr4, chr8, and chr11. Recently, by using the composite interval mapping method, Du et al. [28] uncovered eight major-effect QTL for sesame seed coat color on chromosomes 4, 9, and 12. Moreover, they screened the QTL locations and identified 155 candidate genes associated with sesame seed coat color. However, these candidate genes were not found specifically to be involved in the general phenylpropanoid pathway. Hence, genes involved in biosynthesis and regulation of the sesame seed coat color and how they operate during seed development still needs to be clarified, as only SIN_1016759/PPO, a gene-encoding polyphenol oxidases, had been identified as the regulatory gene that controls seed coat color in sesame [26]. 

RNA-Seq is an advanced approach in transcriptome profiling that uses deep-sequencing technologies. It has become an essential tool for transcriptome-wide analysis of differential gene expression and differential splicing of mRNAs [29]. RNA-seq was successfully used to identify candidate genes for seed coat color in many plants, including *Arabidopsis thaliana* [20], *Brassica rapa* [30], *Brassica napus* [31], and soybean [32]. Additionally, it has been used to detect candidate genes that shaped oil content variation in sesame [33]. In this study, we investigated the transcriptome during the seed development stages at the 5, 8, 11, 14, 17, 20, 23, 26, and 30 days post-anthesis (DPA) of two sesame varieties different in seed coat color “Zhongfengzhi No.1” (white seed) and “Zhongzhi No.33” (black seed). Differentially expressed genes (DEGs) and some candidate genes for sesame seed coat color were identified. This study will facilitate further investigations on seed coat color modulation in sesame and will help to improve sesame varieties quality in the future.

## 2. Materials and Methods

### 2.1. Sesame Varieties 

Two pure lines sesame varieties, “Zhongfengzhi No.1” (white seed) and “Zhongzhi No.33” (black seed) were used in this study. The seeds were given by the National Sesame Medium-term Genebank (Wuhan, China). 

### 2.2. Planting and Sampling

**Figure 2:**
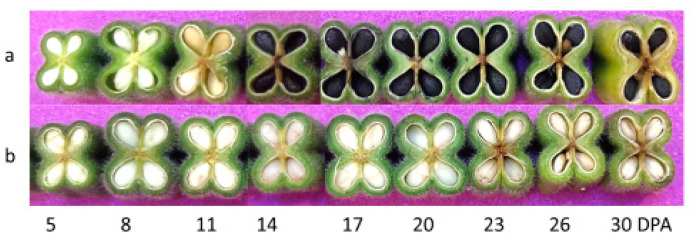
Phenotypes of the seeds at different stages of seed development. (**a**) The black sesame. (**b**) The white sesame.

The two varieties were shown at the experimental station of the Oil Crops Research Institute (OCRI) under identical growth conditions from May to September 2019 in Wuhan, Hubei Province, China, at N 30.57°, E 114.30°, altitude 27 m. Flowers were labeled every three days post-anthesis (DPA). At 5, 8, 11, 14, 17, 20, 23, 26, and 30 DPA, capsules for each variety were sampled from 10 plants (Figure 2), and seeds were separated from the capsules on ice. Different plant seeds were therefore mixed equally and represented samples at 5, 8, 11, 14, 17, 20, 23, 26, and 30 DPA. All samples were prepared in duplicate and were subjected to RNA-seq analysis. 

### 2.3. RNA Extraction and Library Preparation

The seed samples RNA was extracted and sequenced as per Wang et al. [33]. Briefly, for each sample, total RNA was extracted with the TRIzol reagent (Invitrogen Corp., Carlsbad, CA, USA). Then, we used the Oligotex mRNA Midi Kit (Qiagen, Hilden, North Rhine-Westphalia, Germany), to purify the mRNA from the total RNA. The quantity and quality of the mRNA were investigated with Invitrogen Qubit2.0 and Agilent 2100 (Agilent Technologies, Santa Clara, CA, USA). All the mRNAs were then transcribed into double-stranded cDNAs with the SMART cDNA Library Construction kit (Clontech, Mountain View, CA, USA) following the user guide. Finally, appropriate fragments (200 ± 25 bp) were chosen for PCR amplification, and adapters were ligated to the targeted fragments.

### 2.4. Data Generation and Quality Assessment

The libraries of the 36 cDNA generated from the sesame seeds were sequenced for paired-end reads using the Illumina Hiseq 2000 platform (Illumina, Inc., San Diego, CA, USA). The FastQC software (http://www.bioinformatics.babraham.ac.uk/projects/fastqc/) was used to check the reads’ base qualities. We then removed all paired-end reads with more than 5% ambiguous residues (Ns) and those which contained more than 10% bases with a Phred quality score of less than 20. The remaining reads were considered “clean reads” [34]. Finally, 16.3–32.7 million clean reads with a 90 bp length were acquired for each sample. 77.2–85.2% of the clean reads were mapped uniquely to the sesame reference genome after verification of the parameter that no more than one mismatch was accepted in the alignment using TopHat2 [35]. Only 70.4–79.2% of the reads were uniquely mapped to the predicted gene model regions.

### 2.5. Statistical Analysis of Gene Expression

The sample’s genes expression levels were evaluated based on the reads numbers, which were uniquely mapped to the sesame genome sequence [1]. Each gene expression level was then normalized to the number of FPKM (Fragments Per Kilobase of transcript per Million reads) as per Trapnell et al. using Cufflinks 2.0 software [36]. The differentially expressed genes (DEGs) were found out for sesame seed samples following the method described by Chen et al. [37] and Wang et al. [38]. The threshold *p*-value in multiple tests was determined using the Poisson distribution and the false discovery rate (FDR) [39]. The significance of the DEGs was determined using an FDR ≤ 0.01 and absolute value of log2 ratio ≥ 1 [40].

### 2.6. Gene Annotation and Enrichment Analysis

The shared DEGs between the two varieties were annotated with GO terms using Blast2go (https://www.blast2go.com/). The metabolic pathways were identified by performing the KEGG analysis (Kyoto Encyclopedia of Genes and Genomes, http://www.kegg.jp/). Finally, we used the R language packages to analyses the enrichment as per Wang et al. [38]. 

### 2.7. Real-Time Quantitative PCR

The expression profiles of the 17 candidate genes were validated with qRT-PCR, referring to Wang et al. [38] using LightCycler® 480II Real-Time PCR Detection System (Roche Diagnostics, Rotkreuz, Switzerland). Five stages (5, 11, 17, 23, and 30 DPA) seed samples were run in triplicate on the same plate with a negative control that lacked cDNA. The gene actin7 of sesame was used as a positive control. The relative expression levels of the target genes were calculated using the 2^−ΔΔCT^ method [41].

## 3. Results

### 3.1. Phenotypic Variation during Black and White Sesame Seed Development

To identify the stage of black pigmentation formation during seed development, we examined the capsule of “Zhongfengzhi No.1” (white sesame) and “Zhongzhi No.33” (black sesame) developing seeds every three days starting on the 5 DPA. At the early stage (0–8 DPA), there was no difference in the seed coat of the black and white sesame as all the seeds were white (Figure 2). We observed that the black pigment was biosynthesized and accumulated in black sesame seed coat gradually from 8 DPA. The black sesame seeds turned to yellow between 9 and 11 DPA and then black between 12 and 14 DPA. As expected, no color change was noticed in the seed coat of the white sesame during seed formation. 

### 3.2. Seed Transcriptome Difference during White and Black Sesame Seed Coat Development 

**Figure 3:**
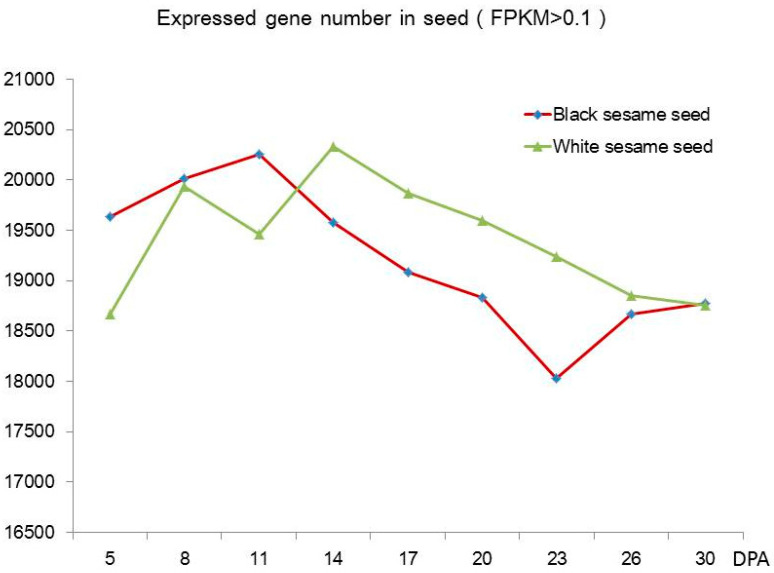
Expressed genes numbers at different DPA in black and white sesame. FPKM: Fragments Per Kilobase of transcript per Million reads.

The black and the white sesame seed samples RNA were sequenced with the Illumina sequencing platform. The unique mapping reads matching the sesame reference genome (version 3) were more than 79.94% for the black sesame and 80.33% for the white sesame (Tables S3 and S4). The black and the white sesame showed similar trends for the expressed genes, with the numbers increased at early stages then decreased. The black sesame reached the maximum expressed gene number with 20,253 DEGs at 11 DPA. The inflection point in white sesame appeared later than the black. The number of expressed genes increased again from 23 DPA to 30 DPA in black sesame (Figure 3).

As the black sesame has started to accumulate pigments or related compounds from 9 DPA, we took 5 and 8 DPA samples as controls to study how the expressed genes change. We compared the other samples to the two points respectively and observed a sharp increase of DEGs number from 8 DPA to 11 DPA especially in the black sesame with 5 DPA as the control (Figures S1 and S2). In the white sesame, no noticeable differences were observed with 5 DPA and 8 DPA as controls. In addition, we found that the down-regulated genes increased more than the up-regulated in both black and white sesames with seed development. 

**Figure 4:**
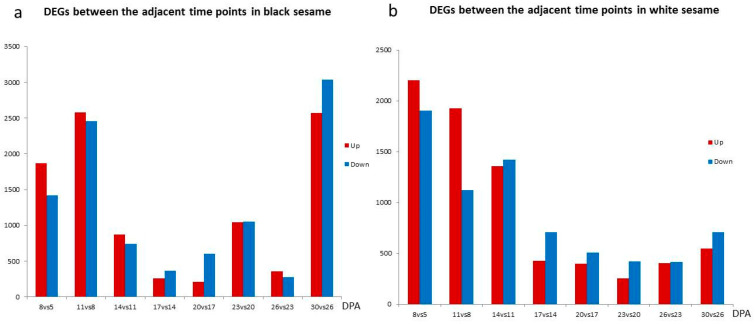
Dynamic change of the differentially expressed genes in black (**a**) and white (**b**) sesame seeds.

We then investigated how the expressed genes change by comparing the seeds to the previous ones dynamically. It showed a significant difference between black and white sesame. In the black sesame, there were more genes up- or down-regulated at the stages of 8, 11, and 30 DPA, and it was increased from 8 to 11 DPA (Figure 4a). In the white sesame, both the up- and down-regulated genes decreased in number from 8 to 23 DPA and then increased slightly (Figure 4b). 

### 3.3. The Differentially Expressed Genes between Black and White Sesame

**Figure 5:**
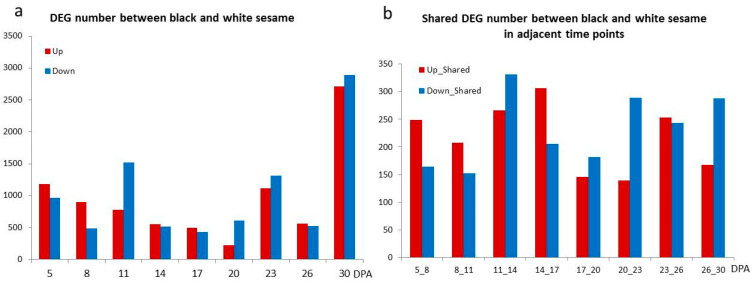
The differentially expressed genes between black and white sesame at different stages. (**a**) DEG number between black and white sesame. (**b**) The shared differentially expressed genes between black and white sesame at two contiguous stages.

We compared the differentially expressed genes between black and white sesames at different stages. The DEG numbers changed with seed development. From 5 to 8 DPA, more up-regulated genes highlighted the early stages, then it decreased to a low level with only 225 up-regulated DEGs at 20 DPA. Many DEGs were observed at the later stage of 30 DPA when the seed reached the maturity stage (Figure 5a). We also studied the shared DEGs by comparing the adjacent two points to reduce the effect of factitious sampling stages. In total, it also showed the black sesame seed had more genes up-regulated at early stages before 17 DPA, and more genes down-regulated at the later stages from 20 to 30 DPA (Figure 5b).

To identify the functional categories of the DEGs between the black and the white sesame, we performed the GO term analysis at different DPA. The DEGs between black and white sesame were enriched into 92 GO terms. Interestingly, only two GO terms were enriched at 5 and 23 DPA. However, several enriched terms in the molecular functions, biological process, and cellular component categories highlighted the 8–20 DPA. Mostly, oxidoreductase activity (GO:0016491), catalytic activity (GO:0003824), iron ion binding (GO:0005506), monooxygenase activity (GO:0004497), electron carrier activity (GO:0009055), heme-binding (GO:0020037) were enriched four or five times from 8 to 20 DPA. At the 30 DPA, the principal GO terms were related to the metabolic process (Table S1). The KEGG pathway analysis assigned the DEGs to 37 metabolic pathways. Among these pathways, the flavonoid biosynthesis and biosynthesis of secondary metabolites were the dominants. Many metabolic pathways including the flavonoid biosynthesis, biosynthesis of secondary metabolites, bisphenol degradation, polycyclic aromatic hydrocarbon degradation, aminobenzoate degradation, limonene, and pinene degradation, flavone, and flavonol biosynthesis, and stilbenoid, diarylheptanoid and gingerol biosynthesis also highlighted the role of 8–20 DPA in black pigment biosynthesis and accumulation. The complete list of the metabolic pathway is provided in (Table S2). To gain insight into the role of flavonoid biosynthesis during black sesame seed coat development, we then mapped the DEGs between black and white sesame from 8 to 23 DPA into the flavonoid biosynthesis pathway. The map indicated that the up-regulated functional KO (KEGG Orthology) increased from 8 to 17 DPA, then decreased from 20 DPA (Figures S6–S11). 

### 3.4. The Candidate Genes Associated with Black Seed Coat Development in Sesame

**Figure 6:**
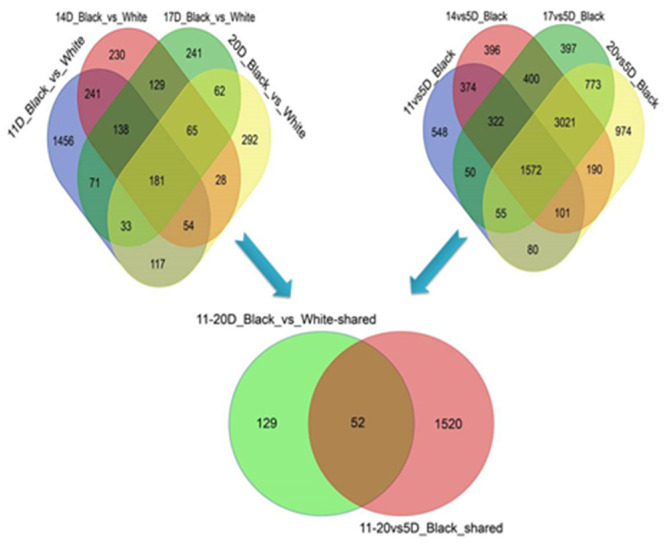
Venn diagram among the common DEGs from 11 to 20 DPA. The gene sets were generated from the shared DEGs between black and white sesame from 11 to 20 DPA and the shared DEGs with 5 DPA as a control in black sesame from 11 to 20 DPA.

The above analysis indicated the seed coat color difference not only exist between black and white sesame but also during seed development in the black sesame. In consideration of the characteristics of seed color changing and genes expression profile, we took the 5 DPA as the initiated control and group 11–20 DPA to identify the candidate genes linked with black pigment biosynthesis in sesame. We identified the shared up- and down-regulated DEGs between 11, 14, 17, and 20 DPA against 5 DPA in the black sesame, and there were 1572 DEGs (Figure 6). Moreover, we analyzed the DEGs and detected that black and white sesames were shared 181 DEGs from 11 to 20 DPA (Figure 6). Finally, we examined the two DEGs sets, and the 52 common genes were selected for further screening (Figure 6).

**Figure 7:**
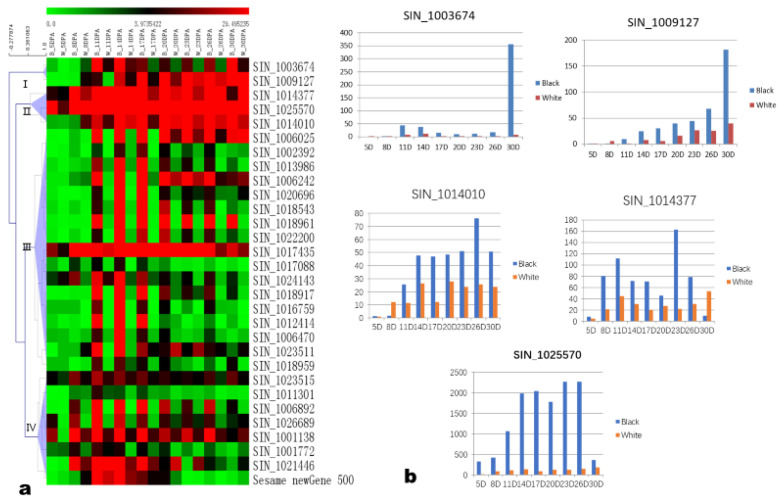
Hierarchical clustering and expression profiles of the 5 DEGs in black and white sesame. (**a**) Hierarchical clustering of the 30 DEGs. (**b**) Expression profiles of subgroup 1 and 2 genes in black and white sesame (only SIN_1025570 was included in the candidate genes list).

From the 52 common DEGs, we removed 12 genes with high FPKM over 100 in white sesame and those less than 5 in both black and white sesame; and 10 conflicting regulated genes between the black and white sesame. The remaining 30 genes were grouped into four subgroups using the hierarchical clustering method (Figure 7a). Subgroup 1 consisted of two genes SIN_1003674 and SIN_1009127. Subgroups 2, 3, and 4 consisted of three, 17, and eight genes, respectively. We then examined the expression profiles of the 30 genes (Figure 7b, Figures S3 and S4) and filtered out 10 genes that had an increased expression level in the white sesame or expressed both in the two sesames with different FPKM. The removed genes include the two genes in subgroup 1, the genes SIN_1014377 and SIN_1014010 in subgroup 2, the gene SIN_1023511 in subgroup 3, and the genes SIN_1001772, SIN_1023515, SIN_1021446, SIN_1011301, and Sesame_newGene_500 in subgroup 4. Finally, the remaining 20 genes, including SIN_1025570 in Subgroup 2; SIN_1006025, SIN_1002392, SIN_1013986, SIN_1006242, SIN_1020696, SIN_1018543, SIN_1018961, SIN_1022200, SIN_1017435, SIN_1017088, SIN_1024143, SIN_1018917, SIN_1016759, SIN_1012414, SIN_1006470, and SIN_1018959 in subgroup 3; SIN_1006892, SIN_1026689, and SIN_1001138 in subgroup 4 were selected as the candidate genes for black seed coat formation in sesame according to their expression patterns and level. These candidate genes are distributed on chromosomes 1–13, except chromosomes 5 and 11. The 20 genes included two chalcone synthase genes SIN_1018961 and SIN_1018959; a dihydroflavonol-4-reductase gene, SIN_1022200; a flavonol synthase/flavanone 3-hydroxylase gene, SIN_1017088; a flavonoid 3’-monooxygenase gene, SIN_1017435; a Myb-related protein gene, SIN_1018543; a glucosyltransferase gene, SIN_1001138, three cytochrome P450 genes, SIN_1006242, SIN_1018917 and SIN_1020696 and the gene SIN_1016759/PPO, which encode a polyphenol oxidase (Table S5). 

### 3.5. qRT-PCR Validation

To verify the expression profiles of the candidate genes identified, we analyzed the RNA-seq assays by qRT-PCR for 16 of them. It showed the same expression patterns confirming the reliability of the RNA-seq data (Figure S5). 

## 4. Discussion

The seed coat is the external protective layer of the seed and develops from the integument initially surrounding the ovule and is maternal in origin [42]. It protects the embryo and endosperm from external factors such as mechanical injuries, desiccation, and infections [13]. Moreover, it helps developing seeds to regulate its metabolism in response to changes in its external environment by transmitting environmental signals to the interior of the seed [13]. In sesame, seed coat color is strongly associated with seed quality [4,6,43]. Therefore, genetic resources on pigmentation, mainly black seed coats, will help to improve the sesame seed quality. In this study, RNA-seq was used to scrutinize transcriptome differences between “Zhongfengzhi No.1” (white seed) and “Zhongzhi No.33” (black seed) at different stages of seed development. DEGs differently regulated during seed coat development were screened, and candidate genes associated with black pigmentation were detected.

We examined the capsule of developing seed of the two varieties and observed that the black sesame seeds were white up to 8 DPA, yellow at 11 DPA, and black at 14 DPA. These results suggested that the biosynthesis and accumulation of black pigment in sesame started from 8 DPA, and 8–14 DPA might be the key period for black seed coat formation in sesame. The phenotype observations were consistent with the RNA-seq data. The maximum number of expressed genes was reached in the black and the white sesame at 11 and 14 DPA, respectively. The comparison of DEGs at different stages against those of 5 DPA showed an increase of DEGs number from 8 to 14 DPA. These findings may suggest a high activity of genes in sesame developing seed between 8 and 14 DPA. Additionally, some genes involved in the seed coat pigments biosynthesis might be initiated at 8 DPA. In the black sesame, more genes were up- or down-regulated at the stages of 8–14 DPA suggesting that this period may play an essential role in the black pigment biosynthesis and accumulation. These results are consistent with the findings of Wei et al. [24,26], who reported that genes expressed highly in sesame seeds from 11 to 20 DPA. Moreover, our results confirmed the pivotal role of later stages in the biosynthesis of nutrients (oil, protein, and lignans) in sesame [33,44,45]. We discovered that more genes were active and up- or down-regulated at the later stages from 23 to 30 DPA in the two varieties, especially in black sesame. Our findings provide the support that in sesame developing seed, early stages play an essential role in seed coat pigments biosynthesis and substrates preparation for nutrients biosynthesis in the later stages. Taken altogether, we thus suggested that black pigment is biosynthesized and accumulates in black sesame developing seed from 8 to 20 DPA mainly. Further examination of several black sesame varieties developing seeds phenotype and transcriptome every day is needed to confirm our results. 

Flavonoids, including anthocyanins and proanthocyanidins (PA), lignin, and melanin, are secondary metabolites that influence seed color in plants [14]. They are derived from the phenylpropanoid pathway and are controlled by a complex regulatory network with multiple transcription factors [18,21]. Some of these genes have been cloned from *Arabidopsis* and many plants. The DEGs between black and white sesame were enriched into 92 GO terms and to 37 metabolic pathways. The flavonoid biosynthesis and biosynthesis of secondary metabolites were the most pathways highlighted. This indicated that flavonoids biosynthesis might be important during black sesame development. The importance of flavonoid biosynthesis during black sesame seed coat development was confirmed by the map of the DEGs between black and white sesame from 8 to 23 DPA into the flavonoid biosynthesis pathway. Flavonoids represent the main secondary metabolites that influence plant seed coat color [46]. Du et al. [28] mapped 14 QTL and uncovered 155 candidate genes for sesame seed coat color that were enriched principally in two pathways, diterpenoid biosynthesis and oxidative phosphorylation. Other studies in sesame detected that two major genes with additive-dominant-epistatic effects plus polygenes with additive-dominant-epistatic effects control the seed coat color, and several other major QTL have been identified [25,27]. Here, we screened the shared DEGs between black and white sesame and identified 20 candidate genes associated with black pigmentation in sesame. The expression differences of these candidate genes were validated by qRT-PCR. The 20 genes included two chalcone synthase (CHS) genes SIN_1018961 and SIN_1018959; a dihydroflavonol-4-reductase (DFR) gene, SIN_1022200; a flavonol synthase/flavanone 3-hydroxylase (F3H) gene, SIN_1017088; a flavonoid 3’-monooxygenase gene, SIN_1017435; a MYB-related protein gene, SIN_1018543; a glucosyltransferase gene, SIN_1001138, and three cytochrome P450 genes, SIN_1006242, SIN_1018917, and SIN_1020696, that may function in the flavonoids pathway. Most of these genes have been well characterized in *A. thaliana* [20]. Chalcone synthase is the first committed enzyme in the biosynthesis of all flavonoids, which function in the phenylpropanoid pathway [20]. It catalyzes the reaction leading to naringenin chalcone formation from p-coumaroyl-CoA and three molecules of malonyl-CoA [47]. DFR is a key regulatory enzyme in the biosynthesis of anthocyanins and PA which catalyzes the reduction of dihydroflavonols (dihydrokaempferol, dihydroquercetin, and dihydromyricetin) to leucoanthocyanidins [48,49]. F3H catalyzes the conversion of flavanones to dihydroflavanols [20]. MYB transcription factor is a complex which is involved in seed developmental and environmental regulation through the activation of flavonoid late biosynthetic genes (LBGs) expression, mainly the expression of DFR [21,50]. MYB genes control the yield of PA in seeds and are also involved in the biosynthesis of lignin in many plants [51]. The glucosyltransferase might be involved in the conversion of leucoanthocyanidins into anthocyanidins [52]. Ahmad et al. [17] reported that cytochrome P450 genes play a crucial regulatory role in osmotic stress tolerance and promote flavonoids accumulation in transgenic *Arabidopsis*. 

SIN_1016759/PPO and SIN_1006025 that encodes polyphenol oxidase and isochorismate synthase, respectively, were also included in the 20 candidate genes. The gene SIN_1016759/PPO had been reported as the candidate gene for black seed coat development in sesame [24,26]. It was also included in the list of sesame seed coat candidate genes reported recently by Du et al. [28]. In plants, browning reactions on seed coat pigments are often induced by the oxidation of phenolic compounds by polyphenol oxidases (PPO) such as laccases and tyrosinases and result in melanin formation mostly [53,54]. Dark seed coat color results from melanogenesis and the oxidation of proanthocyanidins and lignin by PPO [14,16]. Moreover, Wang et al. [12] analyzed the metabolome profile of black and white sesame seeds and found that phenylpropanoid biosynthesis, tyrosine metabolism, and riboflavin metabolism were the main pathways differentially activated between the two seeds and were responsible for the color difference. The above results indicated that flavonoids biosynthesis plays a crucial role in black pigment biosynthesis in sesame. Therefore, we suggest that melanin and/or PA derivates might be responsible for black seed coat color in sesame. Isochorismate synthase converts chorismite, the final product in the shikimate pathway into isochorismate, the precursor of phylloquinone (vitamin K1) and salicylic acid [55]. Salicylic acid has been reported to display an antagonistic interaction with abscisic acid (ABA) in rice [56]. ABA is a plant hormone reported to influence together with R2R3-MYB transcription factor and ethylene, the level of anthocyanin, PA, and lignin in peanut [38]. Otherwise, the gene SIN_1005755/SiNST1 has been previously detected as the major gene that controls lignification in sesame [24]. Our candidate genes do not include any gene related to lignin biosynthesis. These results indicate that the seed coat biosynthesis mechanism in sesame, especially in black sesame, is similar to other plants. All the 20 candidate genes will be targeted in the future for functional genomic study. In addition, the anthocyanin, PA, lignin, and melanin evaluation in sesame seeds with different color is needed to precisely identify the pigment responsible for the dark seed. 

Sesame is especially widely grown for its high-quality nutritional seeds [23]. However, compared with white sesame, black sesame contains less oil, protein, linoleic acid, sesamin, and sesamolin [4,5,6,43,57]. Hence the necessity to improve black sesame quality. The study carried out by Wei et al. [24] revealed that in sesame, the genes PPO and SIN_1005755/SiNST1 are strongly associated with oil, protein, sesamin, and sesamolin content variation in seeds. Furthermore, in sesame aromatic amino acids, L-phenylalanine (Phe) and L-tyrosine (Tyr) are needed for protein biosynthesis and serve as precursors for numerous compounds, including flavonoids, melanin, lignin, lignans, quinones, and condensed tannins [58]. This suggests that a higher yield of flavonoids must be associated with a lower level of lignin and lignans. These amino acids are produced from chorismate, the final product of the shikimate pathway, which involves many other genes [58,59]. Here, we figured out 20 candidate genes associated with black pigment synthesis in sesame, including the genes function in the flavonoids pathway, PPO, isochorismate synthase, and so on. Flavonoids, besides their multiple roles in developmental processes, are anti-oxidative components [60]. Thus, functional analysis coupling with the genetic transformation of these genes simultaneously with other critical genes may be sufficient to improve black sesame quality.

## 5. Conclusions

Overall, our study revealed the transcriptome difference between black and white seed coats during seed development in sesame using RNA-seq analysis. Our results provide valuable information on the complex transcriptome dynamics involved in the control of seed coat color in sesame. The early stages play a crucial role in the biosynthesis and accumulation of the black pigment in *S. indicum* black seed. The phenylpropanoid and flavonoid biosynthetic pathways genes previously identified in other plants are also involved in the formation of seed coat color in sesame. Notably, 20 candidate genes controlling black pigmentation in sesame were identified and will be targeted in future studies for validation. As seed coat color in sesame is strongly associated with seed biochemistry and disease resistance, functional studies (cloning, genome editing, and transformation in sesame or *Arabidopsis*) of these candidate genes will help to understand molecular mechanisms involved in these correlations and for breeding high-quality sesame varieties.

## Supplementary Materials

The following are available online at https://www.mdpi.com/2073-4425/11/12/1399/s1, Additional File 1 which contains: Figure S1. Dynamic change of the differentially expressed genes in black and white sesame with 5 DPA as the control; Figure S2. Dynamic change of the differentially expressed genes in black and white sesame with 8 DPA as the control; Figure S3. The expression profile of the 17 genes in subgroup 3 in the black and white sesame; Figure S4. The expression profile of the eight genes in subgroup 4 in the black and white sesame; Figure S5. qRT-PCR validation of the expression profiles of 16 candidate genes. Additional File 2 which contains: Table S1. Enriched GO terms of DEGs between the black and the white sesame and Table S2. Enriched KEGG pathways of DEGs between the black and the white sesame. Additional File 3, which contains: Table S3. Summary of the transcriptome of black sesame at different stages; Table S4. Summary of the transcriptome of white sesame at different stages and Table S5. Information on the 20 candidate genes. Additional File 4, which contains Figures S6–S11. The map of the DEGs between black and white sesame into the flavonoid pathway from 8 to 23 DPA.

## Additional Data Files

The Raw data of RNA-seq had been submitted to NCBI under the BioProject PRJNA679682.
