# Supplementary material for: Transcriptome Dynamics during Black and White Sesame (Sesamum indicum L.) Seed Development and Identification of Candidate Genes Associated with Black Pigmentation
Source: Genes (Basel). 2020 Nov 25;11(12):1399. doi: 10.3390/genes11121399 (PMC7768470; doi:10.3390/genes11121399)
Supplement: Supplementary file 1 [file genes-11-01399-s002.zip › Additional file 1.pdf]

Additional file 1:

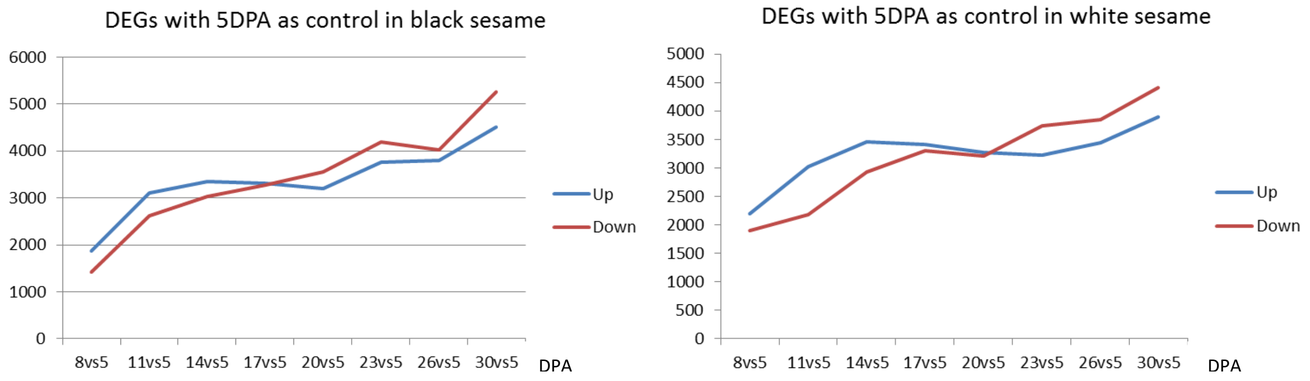

Figure S1. Dynamic change of the differential expressed genes in black and white sesame with 5 DPA as the control.

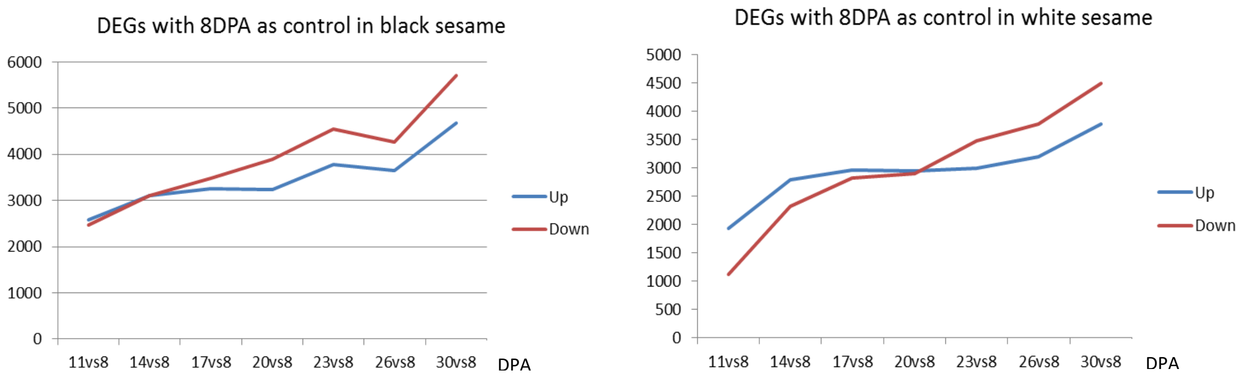

Figure S2. Dynamic change of the differential expressed genes in black and white sesame with 8 DPA as the control

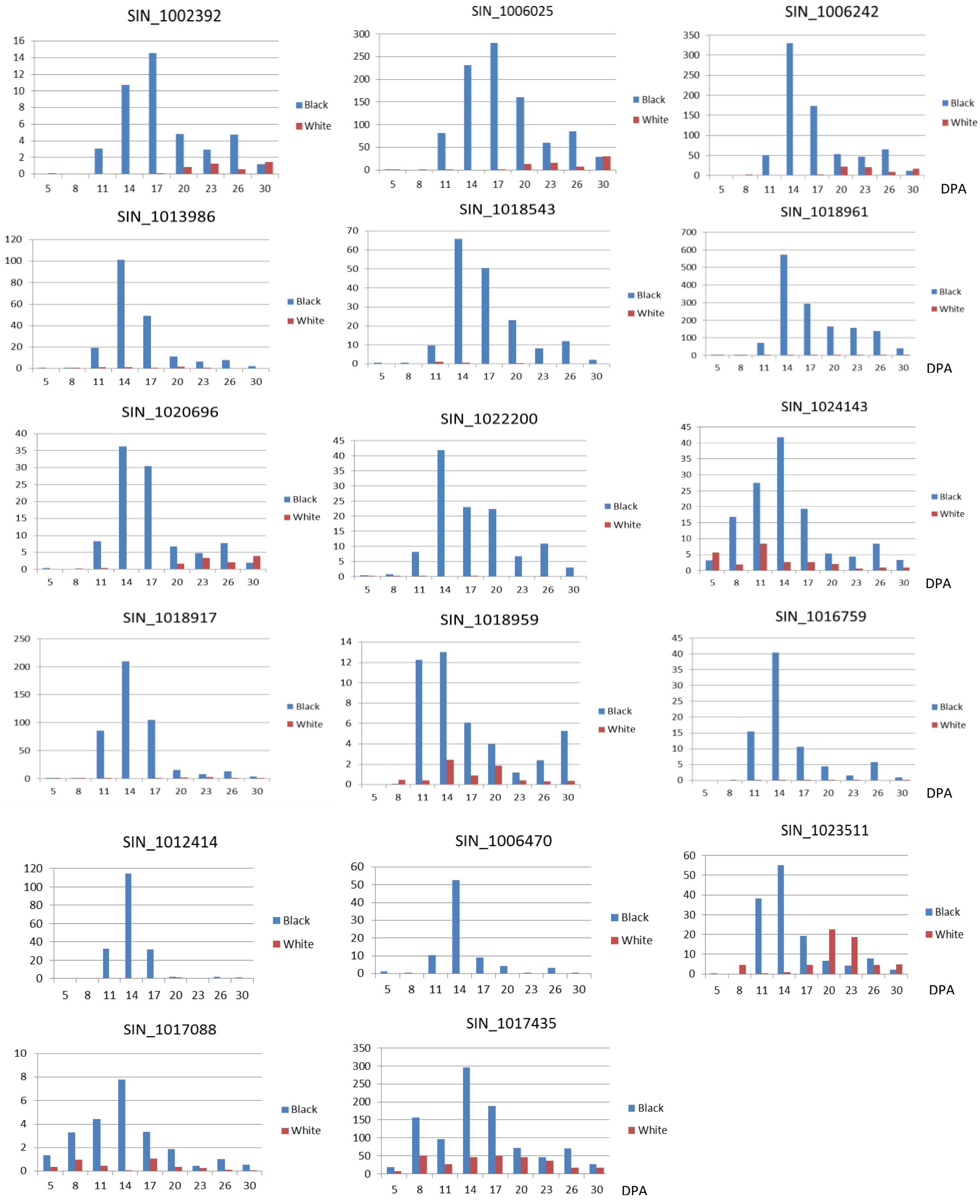

Figure S3. The expression profile of the 17 genes in subgroup3 in the black and white sesame. SIN_1023511 was not included in the candidate genes according to its expression profile.

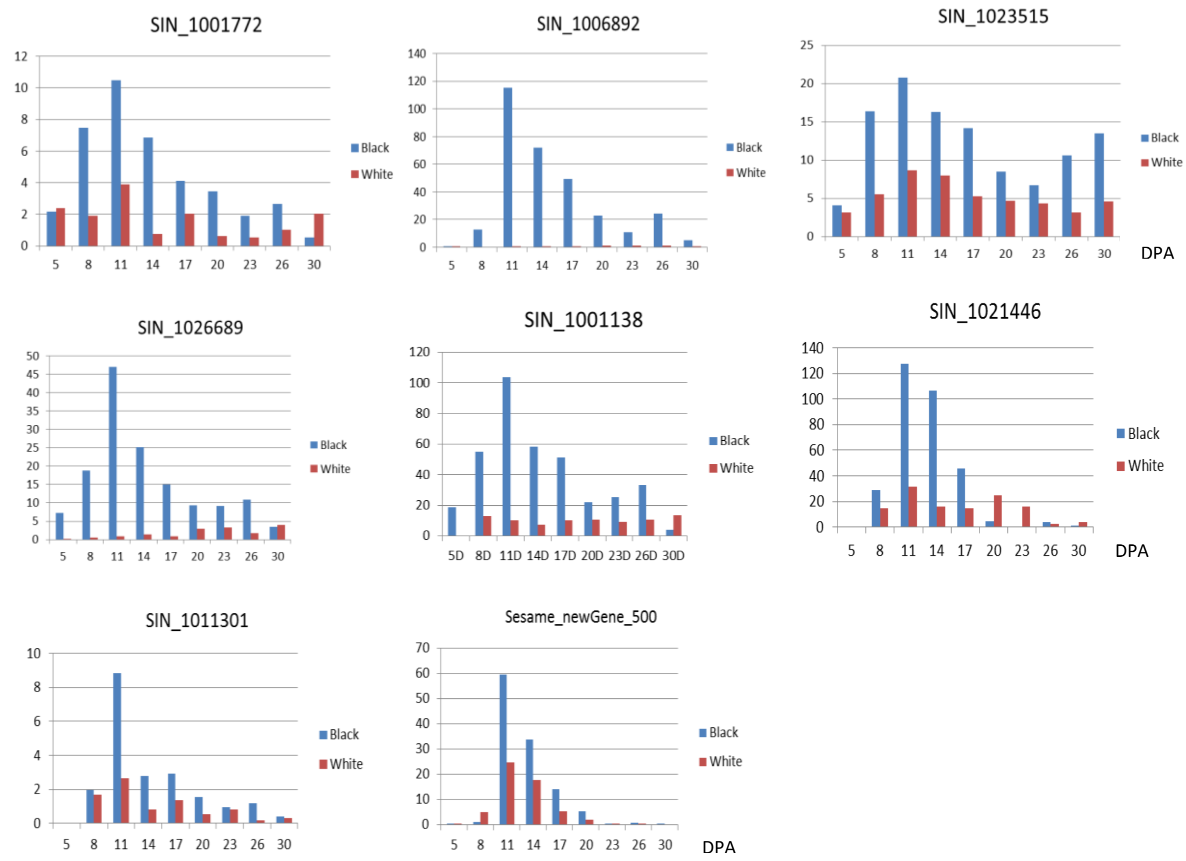

Figure S4. The expression profile of the 8 genes in subgroup 4 in the black and white sesame. SIN_1006892, SIN_1026689 and SIN_1001138 may be included in the candidate genes for their expression profiles.

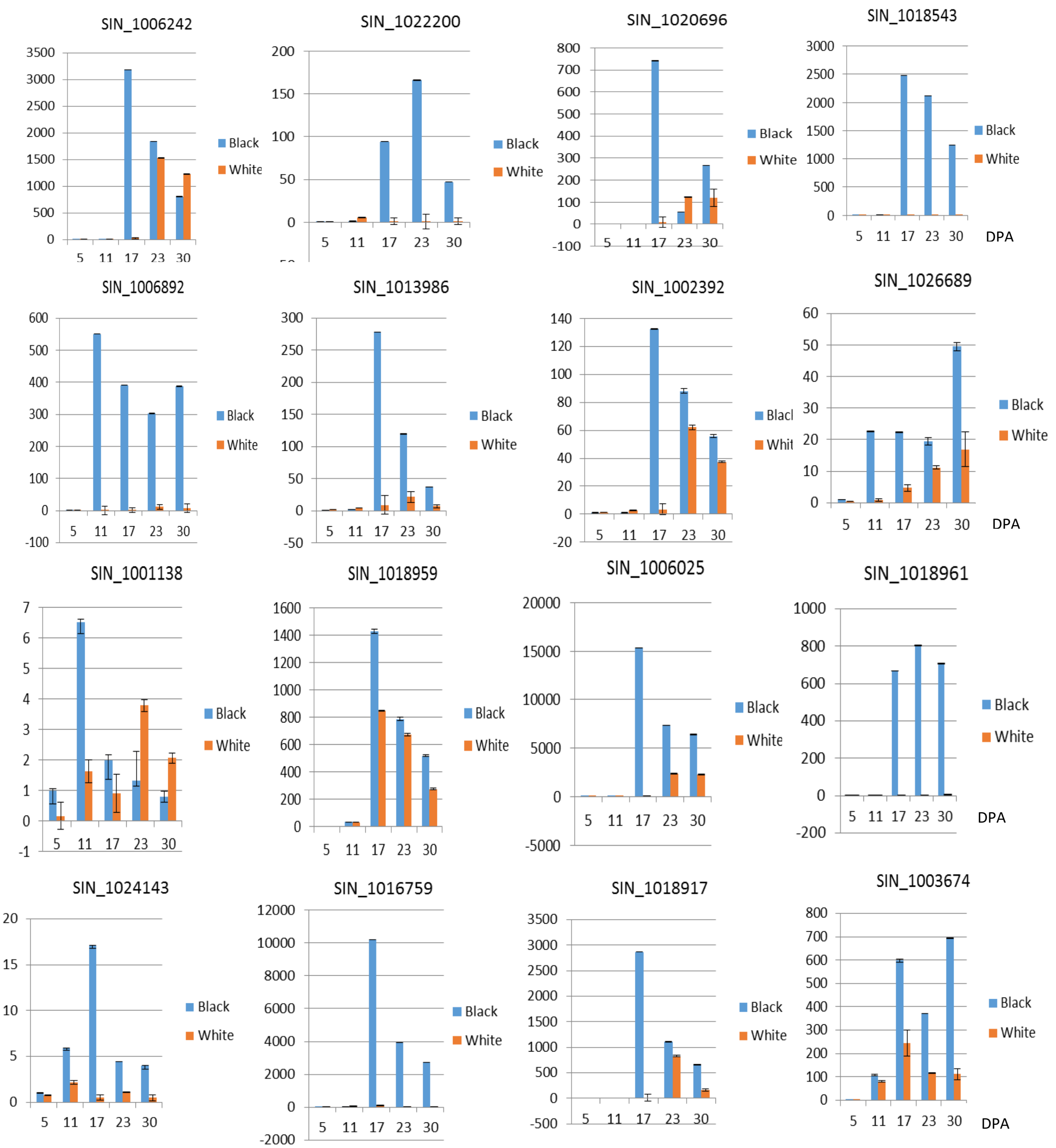

Figure S5. qRT-PCR validation of the expression profiles of 16 candidate genes
