# Supplementary material for: Transcriptome Dynamics during Black and White Sesame (Sesamum indicum L.) Seed Development and Identification of Candidate Genes Associated with Black Pigmentation
Source: Genes (Basel). 2020 Nov 25;11(12):1399. doi: 10.3390/genes11121399 (PMC7768470; doi:10.3390/genes11121399)
Supplement: Supplementary file 1 [file genes-11-01399-s002.zip › Additional file 2.pdf]

Additional file 2

Table S1: Enriched GO terms of DEGs between the black and the white sesame

GO	GO Title	Category	5DPA	8DPA	11DPA	14DPA	17DPA	20DPA	23DPA	26DPA	30DPA	Total
**GO:0016491**	oxidoreductase activity	MF	Y*	Y	Y	Y	Y	Y		Y	Y	8
**GO:0055114**	oxidation-reduction process	BP	Y	Y	Y	Y	Y	Y		Y		7
**GO:0003824**	catalytic activity	MF		Y	Y	Y	Y	Y	Y			6
**GO:0005506**	iron ion binding	MF		Y	Y	Y	Y	Y		Y		6
**GO:0004497**	monooxygenase activity	MF		Y	Y	Y	Y			Y		5
**GO:0009055**	electron carrier activity	MF		Y	Y	Y	Y			Y		5
**GO:0020037**	heme binding	MF		Y	Y	Y	Y			Y		5
**GO:0008152**	metabolic process	BP			Y	Y		Y			Y	4
**GO:0009607**	response to biotic stimulus	BP			Y	Y				Y		3
**GO:0004097**	catechol oxidase activity	MF			Y	Y						2
**GO:0005507**	copper ion binding	MF		Y		Y						2
**GO:0005618**	cell wall	CC			Y	Y						2
**GO:0006629**	lipid metabolic process	BP			Y	Y						2
**GO:0008171**	O-methyltransferase activity	MF		Y		Y						2
**GO:0016757**	transferase activity, transferring glycosyl groups	MF		Y	Y							2
**GO:0016758**	transferase activity, transferring hexosyl groups	MF		Y	Y							2
**GO:0016998**	cell wall macromolecule catabolic process	BP			Y	Y						2
**GO:0048544**	recognition of pollen	BP			Y	Y						2
**GO:0050662**	coenzyme binding	MF		Y		Y						2
**GO:0071554**	cell wall organization or biogenesis	BP			Y	Y						2
**GO:0000003**	reproduction	BP			Y							1

**GO:0003723**	RNA binding	MF								Y	1
**GO:0003735**	structural constituent of ribosome	MF								Y	1
**GO:0004553**	hydrolase activity, hydrolyzing O-glycosyl compounds	MF	Y								1
**GO:0004672**	protein kinase activity	MF		Y							1
**GO:0004674**	protein serine/threonine kinase activity	MF		Y							1
**GO:0004842**	ubiquitin-protein ligase activity	MF		Y							1
**GO:0005198**	structural molecule activity	MF								Y	1
**GO:0005215**	transporter activity	MF				Y					1
**GO:0005529**	sugar binding	MF		Y							1
**GO:0005576**	extracellular region	CC	Y								1
**GO:0005622**	intracellular	CC								Y	1
**GO:0005737**	cytoplasm	CC								Y	1
**GO:0005840**	ribosome	CC								Y	1
**GO:0005975**	carbohydrate metabolic process	BP	Y								1
**GO:0005976**	polysaccharide metabolic process	BP		Y							1
**GO:0006221**	pyrimidine nucleotide biosynthetic process	BP								Y	1
**GO:0006412**	translation	BP								Y	1
**GO:0006468**	protein phosphorylation	BP		Y							1
**GO:0006631**	fatty acid metabolic process	BP		Y							1
**GO:0006855**	drug transmembrane transport	BP			Y						1
**GO:0006857**	oligopeptide transport	BP				Y					1
**GO:0006869**	lipid transport	BP	Y								1
**GO:0008061**	chitin binding	MF		Y							1
**GO:0008194**	UDP-glycosyltransferase activity	MF		Y							1
**GO:0008289**	lipid binding	MF	Y								1
**GO:0009058**	biosynthetic process	BP								Y	1
**GO:0009059**	macromolecule biosynthetic process	BP								Y	1

**GO:0009415**	response to water	BP		Y							1
**GO:0009521**	photosystem	CC						Y			1
**GO:0009523**	photosystem II	CC			Y						1
**GO:0009538**	photosystem I reaction center	CC								Y	1
**GO:0009690**	cytokinin metabolic process	BP	Y								1
**GO:0010467**	gene expression	BP								Y	1
**GO:0012511**	monolayer-surrounded lipid storage body	CC		Y							1
**GO:0016301**	kinase activity	MF		Y							1
**GO:0016310**	phosphorylation	BP		Y							1
**GO:0016567**	protein ubiquitination	BP		Y							1
**GO:0016620**	oxidoreductase activity, acting on the aldehyde or oxo group of donors, NAD or NADP as acceptor	MF	Y								1
**GO:0016740**	transferase activity	MF		Y							1
**GO:0016773**	phosphotransferase activity, alcohol group as acceptor	MF		Y							1
**GO:0016798**	hydrolase activity, acting on glycosyl bonds	MF	Y								1
**GO:0016841**	ammonia-lyase activity	MF			Y						1
**GO:0016903**	oxidoreductase activity, acting on the aldehyde or oxo group of donors	MF	Y								1
**GO:0019139**	cytokinin dehydrogenase activity	MF	Y								1
**GO:0030246**	carbohydrate binding	MF		Y							1
**GO:0030312**	external encapsulating structure	CC		Y							1
**GO:0030529**	ribonucleoprotein complex	CC								Y	1
**GO:0032501**	multicellular organismal process	BP		Y							1
**GO:0032991**	macromolecular complex	CC								Y	1
**GO:0033897**	ribonuclease T2 activity	MF	Y								1

**GO:0034645**	cellular macromolecule biosynthetic process	BP								Y	1
**GO:0042026**	protein refolding	BP								Y	1
**GO:0043169**	cation binding	MF			Y						1
**GO:0043229**	intracellular organelle	CC								Y	1
**GO:0043232**	intracellular non-membrane-bounded organelle	CC								Y	1
**GO:0044036**	cell wall macromolecule metabolic process	BP		Y							1
**GO:0044237**	cellular metabolic process	BP								Y	1
**GO:0044238**	primary metabolic process	BP								Y	1
**GO:0044249**	cellular biosynthetic process	BP								Y	1
**GO:0044267**	cellular protein metabolic process	BP								Y	1
**GO:0044424**	intracellular part	CC								Y	1
**GO:0044444**	cytoplasmic part	CC								Y	1
**GO:0045735**	nutrient reservoir activity	MF	Y								1
**GO:0046872**	metal ion binding	MF			Y						1
**GO:0046906**	tetrapyrrole binding	MF				Y					1
**GO:0046914**	transition metal ion binding	MF			Y						1
**GO:0048037**	cofactor binding	MF			Y						1
**GO:0050660**	flavin adenine dinucleotide binding	MF	Y								1
**GO:0052689**	carboxylic ester hydrolase activity	MF		Y							1
**GO:0071702**	organic substance transport	BP	Y								1
**GO:0072528**	pyrimidine-containing compound biosynthetic process	BP								Y	1

Y*： Yes

Table S2: Enriched KEGG pathways of DEGs between the black and the white sesame

MapID	MapTitle	5DPA	8DPA	11DPA	14DPA	17DPA	20DPA	23DPA	26DPA	30DPA	Total
**map00941**	Flavonoid biosynthesis		Y	Y	Y	Y	Y	Y	Y		7
**map01110**	Biosynthesis of secondary metabolites	Y	Y	Y	Y	Y	Y		Y		7
**map01100**	Metabolic pathways	Y	Y	Y	Y	Y			Y		6
**map00363**	Bisphenol degradation		Y	Y	Y	Y			Y		5
**map00624**	Polycyclic aromatic hydrocarbon degradation		Y	Y	Y	Y			Y		5
**map00627**	Aminobenzoate degradation		Y	Y	Y	Y			Y		5
**map00903**	Limonene and pinene degradation		Y	Y	Y	Y			Y		5
**map00944**	Flavone and flavonol biosynthesis		Y	Y	Y	Y			Y		5
**map00945**	Stilbenoid, diarylheptanoid and gingerol biosynthesis		Y	Y	Y	Y			Y		5
**map04976**	Bile secretion			Y	Y			Y	Y		4
**map00904**	Diterpenoid biosynthesis			Y		Y			Y		3
**map00906**	Carotenoid biosynthesis			Y	Y		Y				3
**map00940**	Phenylpropanoid biosynthesis			Y	Y	Y					3
**map01120**	Microbial metabolism in diverse environments				Y	Y			Y		3
**map02010**	ABC transporters				Y			Y	Y		3
**map00196**	Photosynthesis - antenna proteins	Y						Y			2
**map00360**	Phenylalanine metabolism			Y	Y						2
**map00592**	alpha-Linolenic acid metabolism	Y		Y							2
**map00010**	Glycolysis / Gluconeogenesis	Y									1
**map00040**	Pentose and glucuronate interconversions			Y							1
**map00053**	Ascorbate and aldarate metabolism				Y						1

**map00270**	Cysteine and methionine metabolism			Y	1
**map00950**	Isoquinoline alkaloid biosynthesis		Y		1
**map01040**	Biosynthesis of unsaturated fatty acids		Y		1
**map03010**	Ribosome			Y	1
**map04075**	Plant hormone signal transduction	Y			1
**map04210**	Apoptosis	Y			1
**map04620**	Toll-like receptor signaling pathway	Y			1
**map04626**	Plant-pathogen interaction	Y			1
**map04722**	Neurotrophin signaling pathway	Y			1
**map04740**	Olfactory transduction	Y			1
**map04916**	Melanogenesis	Y			1
**map04970**	Salivary secretion	Y			1
**map04971**	Gastric acid secretion	Y			1
**map04974**	Protein digestion and absorption	Y			1
**map05140**	Leishmaniasis	Y			1
**map05145**	Toxoplasmosis	Y			1

Y*： Yes
