# Supplementary material for: Transcriptome Dynamics during Black and White Sesame (Sesamum indicum L.) Seed Development and Identification of Candidate Genes Associated with Black Pigmentation
Source: Genes (Basel). 2020 Nov 25;11(12):1399. doi: 10.3390/genes11121399 (PMC7768470; doi:10.3390/genes11121399)
Supplement: Supplementary file 1 [file genes-11-01399-s002.zip › Additional file 3.pdf]

Additional file 3

Table S3: Summary of the transcriptome of black sesame at different stages

DPA	5 DPA	8 DPA	11 DPA	14 DPA	17 DPA	20 DPA	23 DPA	26 DPA	30 DPA
Obtained Clean Pair-end Reads (million)	82.54831	98.69437	91.1944	78.81151	89.1253	97.992	90.52644	78.857902	79.5351
Obtained Clean Bases (Mbp)	10401.09	12435.49	11490.5	9930.251	11229.8	12347	11406.33	9936.0957	10021.4
Mapped Reads (million)	70.13013	87.31255	77.499	66.51305	75.0768	82.1375	76.8366	66.735251	67.5291
Mapped Reads(%)	84.785	88.465	84.96	84.395	84.185	83.82	85.045	84.805	84.97
Unique Mapped Reads (million)	66.72167	83.81085	73.8241	64.82752	72.0286	78.3307	74.32997	65.257813	65.7833
Unique Mapped Reads(%)	80.465	84.935	80.85	82.255	80.795	79.945	82.245	82.98	82.78

Table S4: Summary of the transcriptome of white sesame at different stages

DPA	5 DPA	8 DPA	11 DPA	14 DPA	17 DPA	20 DPA	23 DPA	26 DPA	30 DPA
Obtained Clean Pair-end Reads (million)	90.39882	118.4011	111.655	83.84413	123.589	90.2825	102.4909	89.889964	86.5798
Obtained Clean Bases (Mbp)	11390.25	14064.96	14068.5	10564.36	16425.8	11375.6	12913.85	11326.135	10909.1
Mapped Reads (million)	78.00359	102.3412	95.9727	71.93774	104.942	77.2522	86.77828	75.435828	72.2751
Mapped Reads(%)	86.275	86.4	85.96	85.77	84.93	85.585	84.66	83.885	83.47
Unique Mapped Reads (million)	74.59041	96.28342	91.026	69.03757	99.1779	73.4462	84.72037	72.438361	70.3773
Unique Mapped Reads(%)	82.54	81.49	81.515	82.355	80.335	81.335	82.67	80.68	81.28

Table S5: Information on the 20 candidate genes

Chr_V2	Gene ID	NCBI annotation-blast	swissprot	trembl	IPR
**chr7**	SIN_1001138	crocetin glucosyltransferase, chloroplastic-like [Sesamum indicum]	U75C1_ARATH UDP-glycosyltransferase 75C1 OS=Arabidopsis thaliana GN=UGT75C1 PE=2 SV=2	Q8RU71_TOBAC Glucosyltransferase OS=Nicotiana tabacum GN=NtGT2 PE=2 SV=1	IPR002213; UDP-glucuronosyl/UDP-glucosyltransferase
**chr2**	SIN_1002392	probable carotenoid cleavage dioxygenase 4, chloroplastic[Sesamum indicum]	CCD4_ARATH Probable carotenoid cleavage dioxygenase 4, chloroplastic OS=Arabidopsis thaliana GN=CCD4 PE=1 SV=1	Q2PHF8_LACSA Carotenoid cleavage dioxygenase 1 OS=Lactuca sativa GN=LsCCD1 PE=2 SV=1	IPR004294; Carotenoid oxygenase
**chr8**	SIN_1006025	isochorismate synthase, chloroplastic [Sesamum indicum]	ICS_CATRO Isochorismate synthase, chloroplastic OS=Catharanthus roseus PE=1 SV=1	B9R951_RICCO Isochorismate synthase, putative OS=Ricinus communis GN=RCOM_1513750 PE=4 SV=1	IPR015890; Chorismate binding, C-terminal
**chr4**	SIN_1006242	cytochrome P450 93A3-like [Sesamum indicum]	C93A1_SOYBN Cytochrome P450 93A1 OS=Glycine max GN=CYP93A1 PE=2 SV=1	B9II24_POPTR Cytochrome P450 OS=Populus trichocarpa GN=CYP93A4 PE=3 SV=1	IPR001128; Cytochrome P450 IPR002401; Cytochrome P450, E-class, group I IPR017972; Cytochrome P450, conserved site
**chr12**	SIN_1006892	uncharacterized protein LOC105175232 [Sesamum indicum]	NA	C6T3E9_SOYBN Putative uncharacterized protein OS=Glycine max PE=2 SV=1	IPR006041; Pollen Ole e 1 allergen/extensin
**chr1**	SIN_1013986	ferruginol synthase-like [Sesamum indicum]	C76C1_ARATH Cytochrome P450 76C1 OS=Arabidopsis thaliana GN=CYP76C1 PE=2 SV=1	Q2PF19_VERHY Cytochrome P450 OS=Verbena hybrida PE=2 SV=1	IPR001128; Cytochrome P450 IPR002401; Cytochrome P450, E-class, group I IPR017972; Cytochrome P450, conserved site

**chr4**	SIN_1016759	polyphenol oxidase I, chloroplastic-like [Sesamum indicum]	PPO_MALDO Polyphenol oxidase, chloroplastic OS=Malus domestica PE=2 SV=1	B9VS06_9ERIC Polyphenoloxidase OS=Camellia nitidissima PE=2 SV=1	IPR002227; Tyrosinase IPR022739; Polyphenol oxidase, central domain IPR022740; Polyphenol oxidase, C-terminal
**chr6**	SIN_1018543	transcription factor WER-like [Sesamum indicum]	MYB1_MAIZE Myb-related protein Zm1 OS=Zea mays PE=2 SV=1	Q7Y0W6_PERFR MYBC05 OS=Perilla frutescens var. crispa GN=mybc05 PE=2 SV=1	IPR001005; SANT domain, DNA binding IPR014778; Myb, DNA-binding IPR017877; MYB-like IPR017930; Transcription regulator HTH, Myb-type, DNA-binding
**chr10**	SIN_1018917	cytochrome P450 71A1-like [Sesamum indicum]	C83B1_ARATH Cytochrome P450 83B1 OS=Arabidopsis thaliana GN=CYP83B1 PE=1 SV=1	E0CPK7_VITVI Whole genome shotgun sequence of line PN40024, scaffold_1.assembly12x (Fragment) OS=Vitis vinifera GN=VIT_00009854001 PE=4 SV=1	IPR001128; Cytochrome P450 IPR002401; Cytochrome P450, E-class, group I IPR017972; Cytochrome P450, conserved site
**chr10**	SIN_1018959	chalcone synthase-like [Sesamum indicum]	CHSY_PERFR Chalcone synthase OS=Perilla frutescens GN=CHS PE=2 SV=1	A5A369_SOLSC Chalcone synthase OS=Solenostemon scutellarioides PE=2 SV=1	IPR001099; Chalcone/stilbene synthase, N-terminal IPR012328; Chalcone/stilbene synthase, C-terminal
**chr10**	SIN_1018961	chalcone synthase [Sesamum indicum]	CHSY_ANTMA Chalcone synthase OS=Antirrhinum majus GN=CHS PE=3 SV=1	Q2WBN0_9LAMI Chalcone synthase OS=Misopates orontium GN=chs PE=2 SV=1	IPR001099; Chalcone/stilbene synthase, N-terminal IPR012328; Chalcone/stilbene synthase, C-terminal IPR018088; Chalcone/stilbene synthase, active site
**chr6**	SIN_1020696	cytochrome P450 71D95-like [Sesamum indicum]	C71DF_MENPI Cytochrome P450 71D15 OS=Mentha piperita GN=CYP71D15 PE=1 SV=1	D2KC84_SCODU Cytochrome P450 CYP71D177 OS=Scoparia dulcis GN=CYP6 PE=2 SV=1	IPR001128; Cytochrome P450 IPR002401; Cytochrome P450, E-class, group I IPR017972; Cytochrome P450, conserved site

**chr12**	SIN_1022200	LOW QUALITY PROTEIN: dihydroflavonol 4-reductase [Sesamum indicum]	DFRA_ANTMA Dihydroflavonol-4-reductase OS=Antirrhinum majus GN=DFRA PE=2 SV=1	O04113_PERFR Dihydroflavonol 4-reductase OS=Perilla frutescens PE=2 SV=1	IPR001509; NAD-dependent epimerase/dehydratase
**chr9**	SIN_1024143	MLO-like protein 6 [Sesamum indicum]	MLO6_ARATH MLO-like protein 6 OS=Arabidopsis thaliana GN=MLO6 PE=2 SV=2	B9HQB2_POPTR Predicted protein OS=Populus trichocarpa GN=POPTRDRAFT_558261 PE=4 SV=1	IPR004326; Mlo-related protein
**chr8**	SIN_1026689	WAT1-related protein At3g28050-like [Sesamum indicum]	5NG4_PINTA Auxin-induced protein 5NG4 OS=Pinus taeda PE=2 SV=1	B9HJR3_POPTR Predicted protein OS=Populus trichocarpa GN=POPTRDRAFT_766289 PE=4 SV=1	IPR000620; Drug/metabolite transporter
**chr13**	SIN_1006470	NA	GSTF7_ARATH Glutathione S-transferase OS=Arabidopsis thaliana GN=At3g03190 PE=2 SV=1	B1B5E6_PERFR Glutathione S-transferase OS=Perilla frutescens var. crispa GN=gst1 PE=2 SV=1	IPR004045; Glutathione S-transferase, N-terminal IPR004046; Glutathione S-transferase, C-terminal IPR017933; Glutathione S-transferase/chloride channel, C-terminal
**chr6**	SIN_1012414	NA	KSA_PEA Ent-copalyl diphosphate synthase, chloroplastic OS=Pisum sativum PE=2 SV=1	Q59JI6_SCODU Putative copalyl diphosphate synthase OS=Scoparia dulcis GN=cps PE=2 SV=1	IPR001906; Terpene synthase-like IPR005630; Terpene synthase, metal-binding domain
**chr2**	SIN_1017088	NA	FLS_PETHY Flavonol synthase/flavanone 3-hydroxylase OS=Petunia hybrida GN=FL PE=2 SV=1	Q2XP01_ANTMA Flavonol synthase OS=Antirrhinum majus PE=2 SV=1	IPR005123; Oxoglutarate/iron-dependent oxygenase

**chr3**	SIN_1017435	NA	F3PH_PETHY Flavonoid 3'-monooxygenase OS=Petunia hybrida GN=CYP75B2 PE=2 SV=1	Q2XP00_ANTMA Flavonoid-3'-hydroxylase OS=Antirrhinum majus PE=2 SV=1	IPR001128; Cytochrome P450 IPR002401; Cytochrome P450, E-class, group I IPR017972; Cytochrome P450, conserved site
**chr9**	SIN_1025570	NA	NA	NA	NA
