# Supplementary material for: Transcriptome Dynamics during Black and White Sesame (Sesamum indicum L.) Seed Development and Identification of Candidate Genes Associated with Black Pigmentation
Source: Genes (Basel). 2020 Nov 25;11(12):1399. doi: 10.3390/genes11121399 (PMC7768470; doi:10.3390/genes11121399)
Supplement: Supplementary file 1 [file genes-11-01399-s002.zip › Additional file 4.pdf]

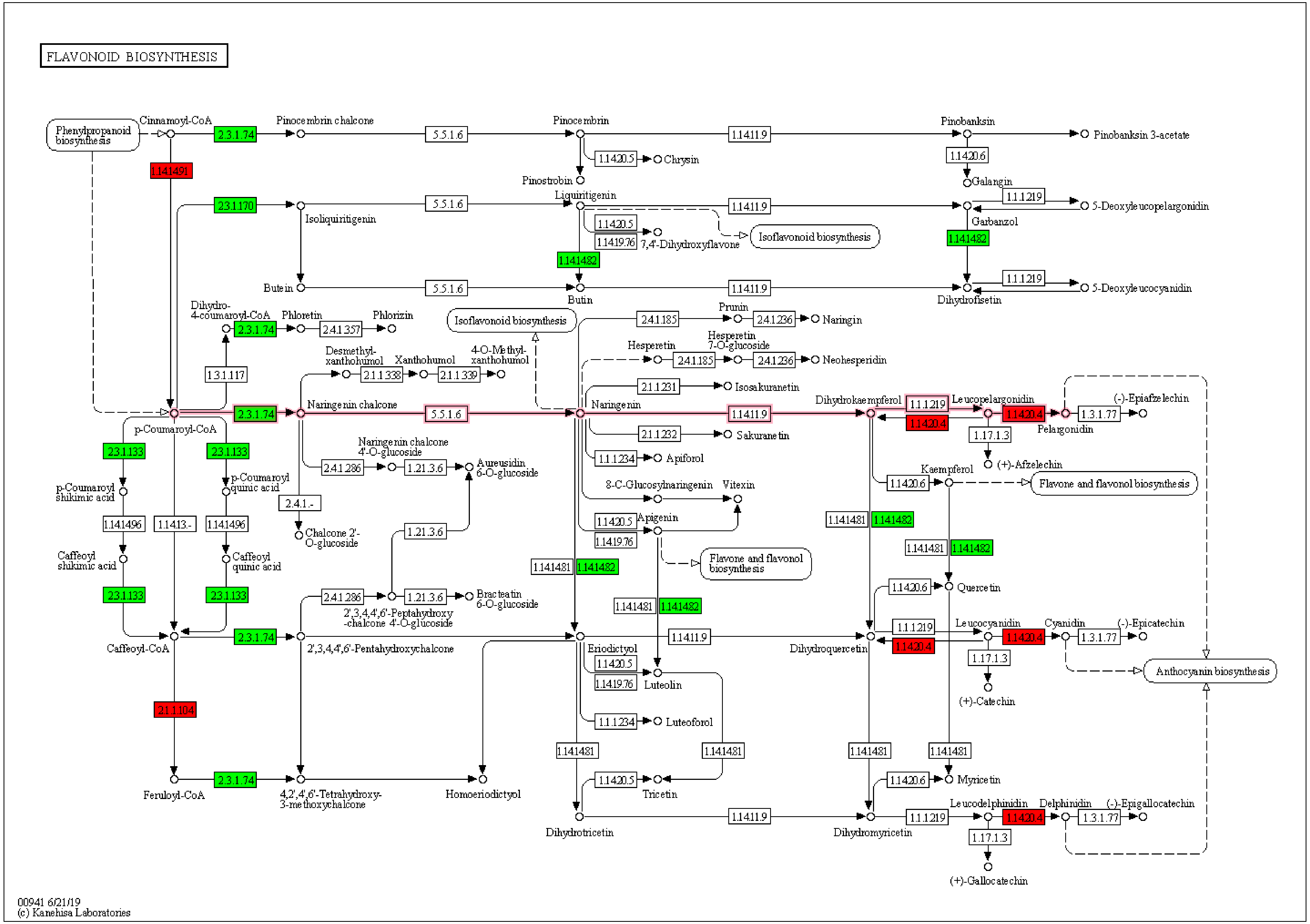

Red bar: up-regulated genes in black sesame

Green bar: down-regulated genes in black sesame

Figure S6. The map of the DEGs between black and white sesame into the flavonoid pathway at 8 DPA.

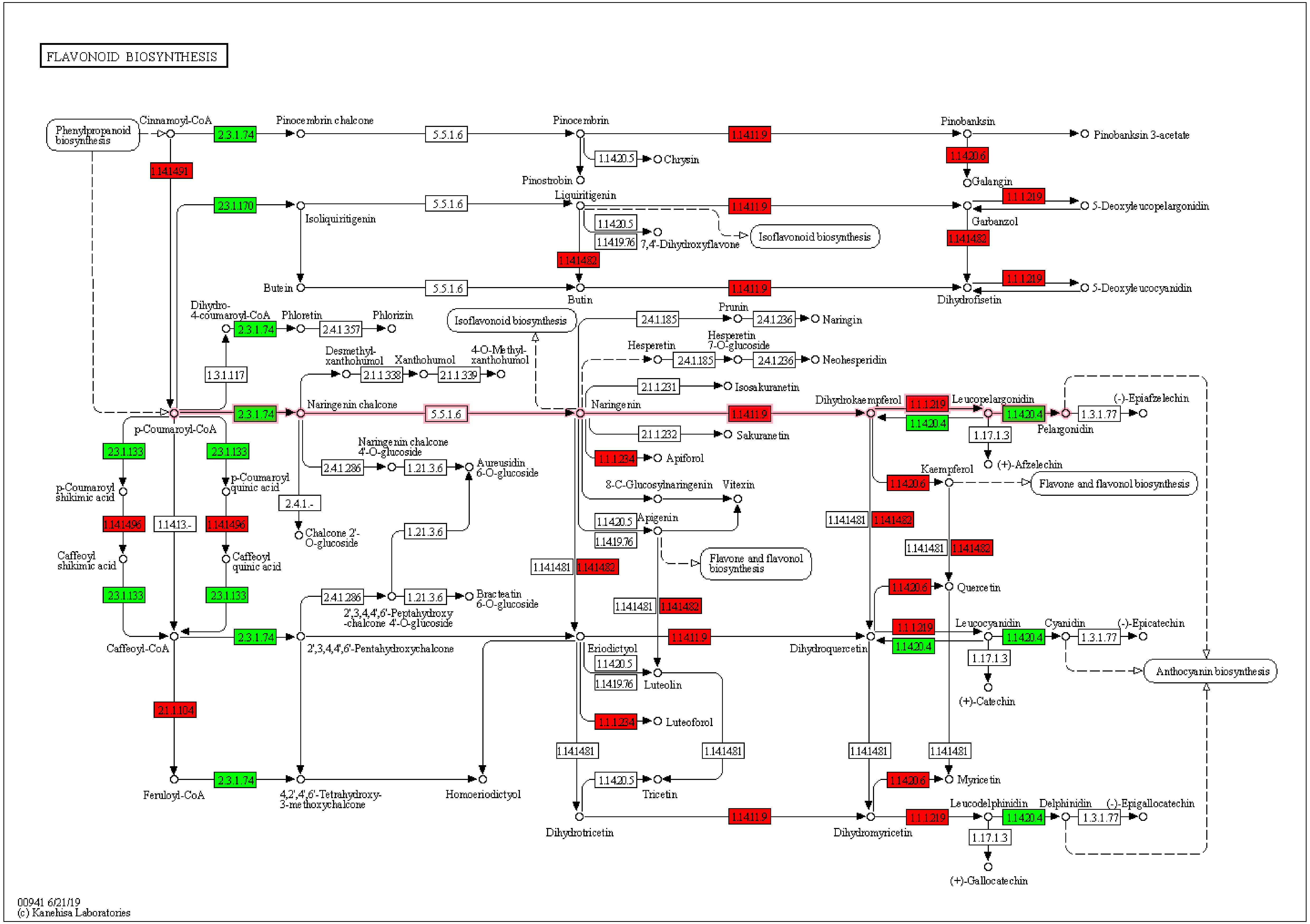

Red bar: up-regulated genes in black sesame

Green bar: down-regulated genes in black sesame

Figure S7. The map of the DEGs between black and white sesame into the flavonoid pathway at 11 DPA.

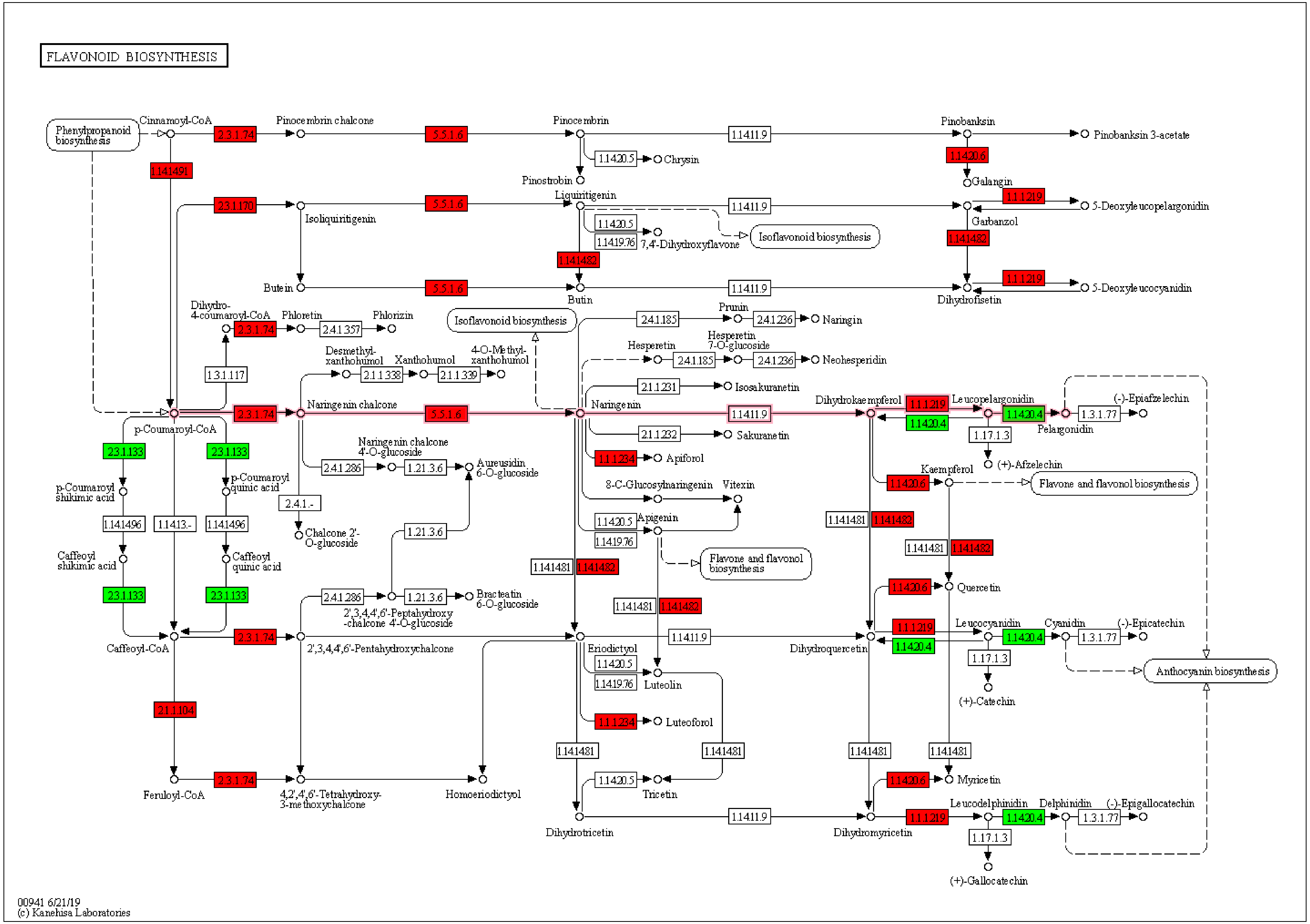

Red bar: up-regulated genes in black sesame

Green bar: down-regulated genes in black sesame

Figure S8. The map of the DEGs between black and white sesame into the flavonoid pathway at 14 DPA.

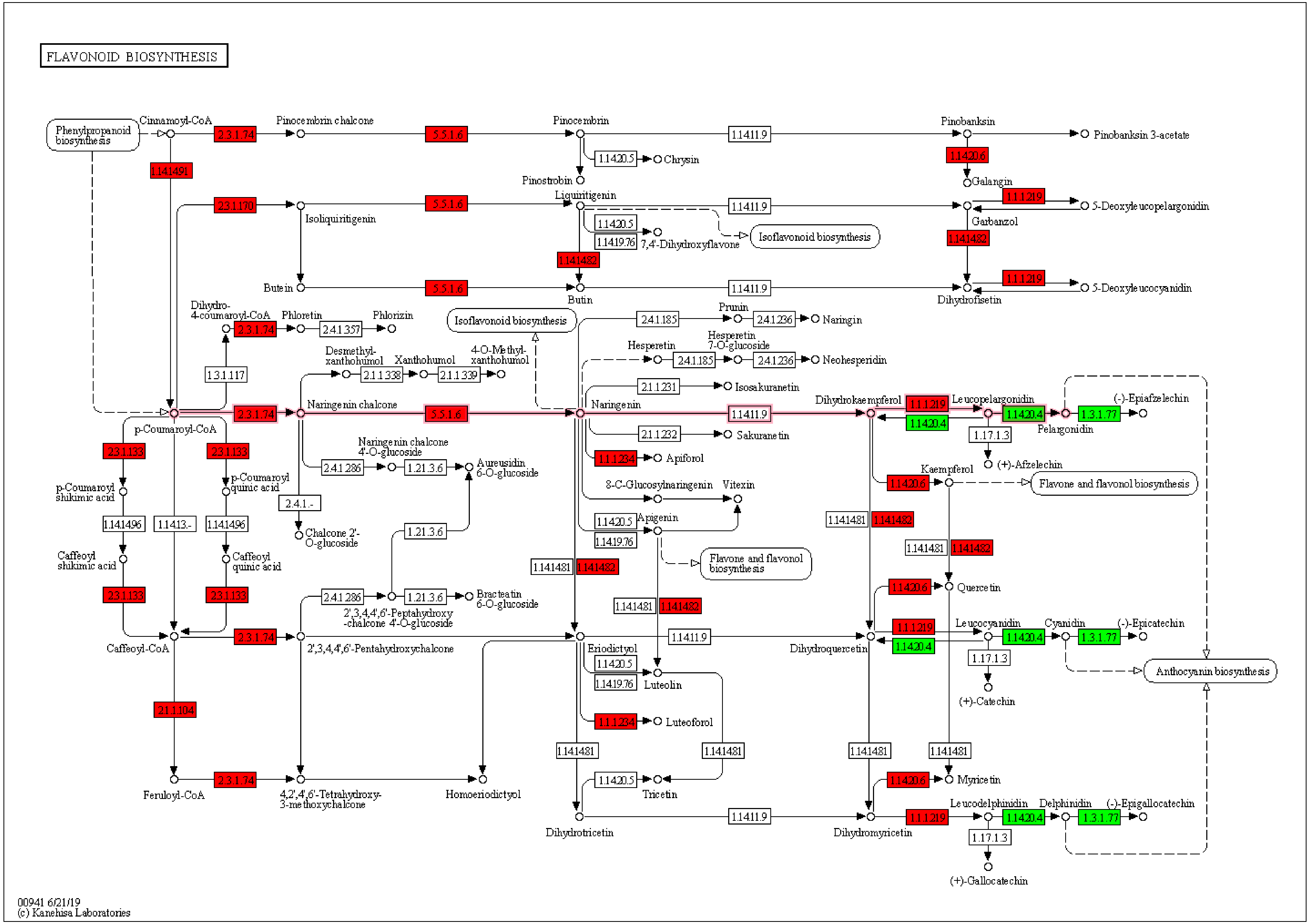

Red bar: up-regulated genes in black sesame

Green bar: down-regulated genes in black sesame

Figure S9. The map of the DEGs between black and white sesame into the flavonoid pathway at 17 DPA.

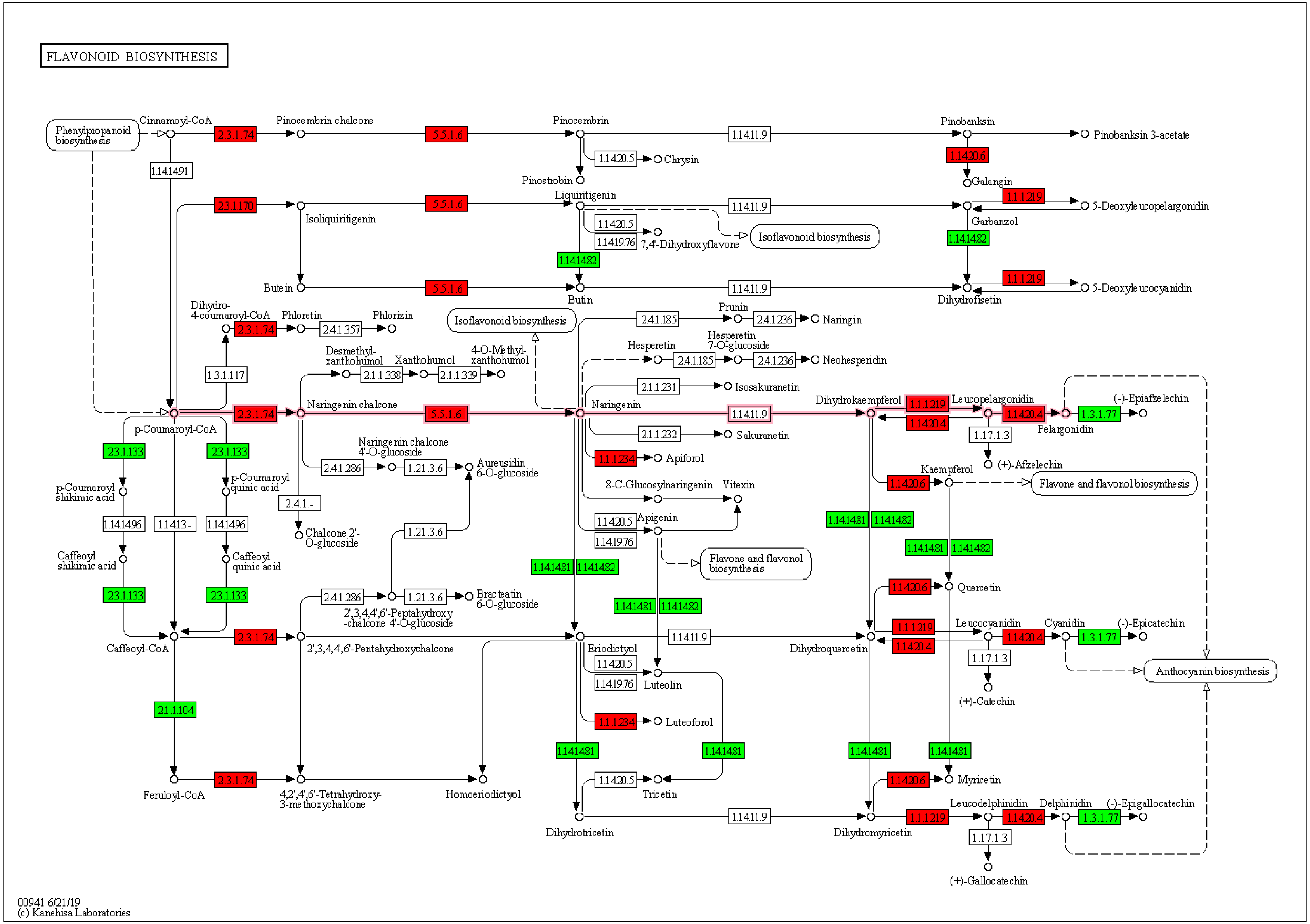

Red bar: up-regulated genes in black sesame

Green bar: down-regulated genes in black sesame

Figure S10. The map of the DEGs between black and white sesame into the flavonoid pathway at 20 DPA.

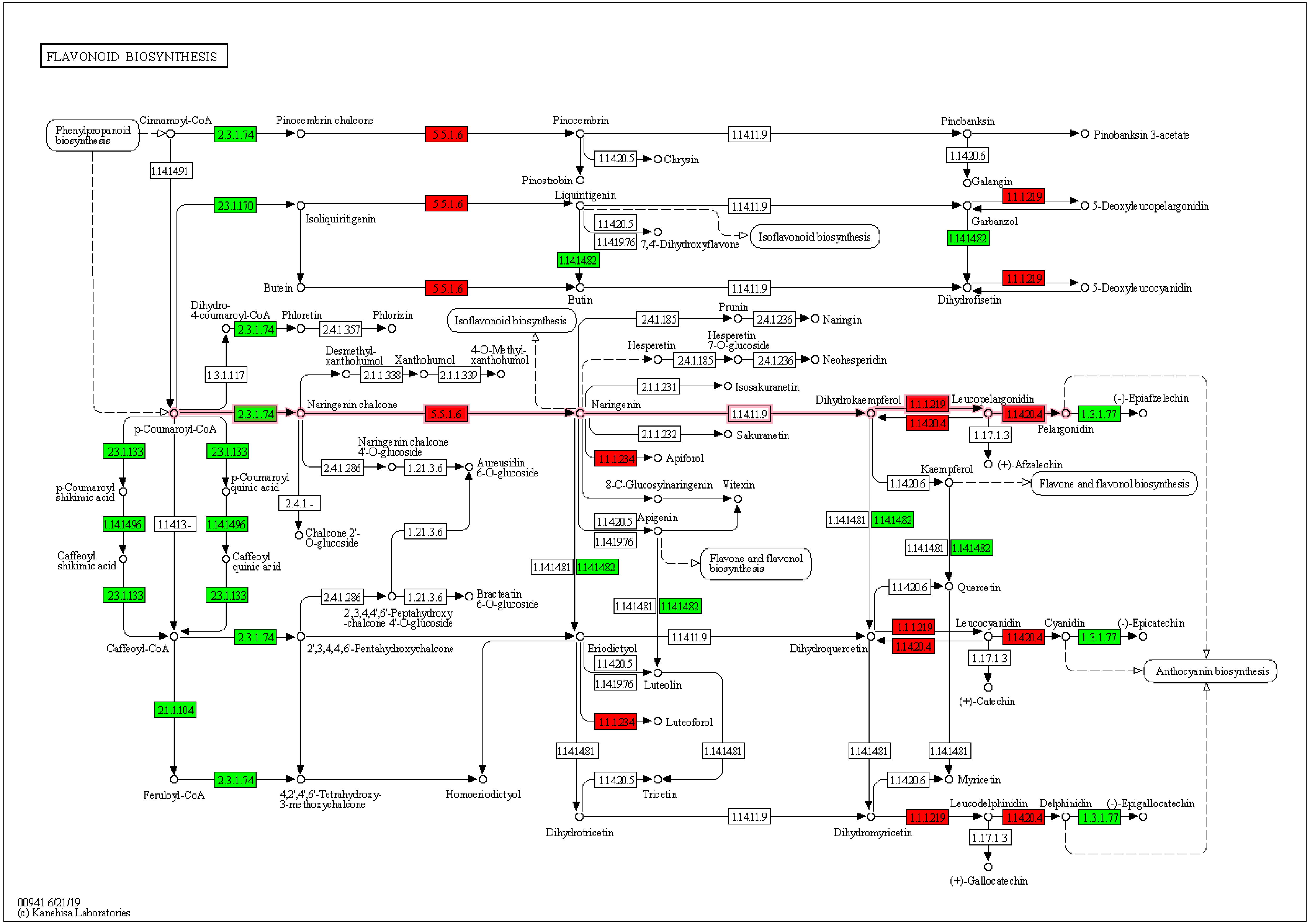

Red bar: up-regulated genes in black sesame

Green bar: down-regulated genes in black sesame

Figure S11. The map of the DEGs between black and white sesame into the flavonoid pathway at 23 DPA.
